# Vitamin D Metabolic Pathway Genes Polymorphisms and Their Methylation Levels in Association With Rheumatoid Arthritis

**DOI:** 10.3389/fimmu.2021.731565

**Published:** 2021-12-02

**Authors:** Tian-Ping Zhang, Hong-Miao Li, Qian Huang, Li Wang, Xiao-Mei Li

**Affiliations:** ^1^ Department of Rheumatology and Immunology, The First Affiliated Hospital of USTC, Division of Life Sciences and Medicine, University of Science and Technology of China, Hefei, China; ^2^ Department of Epidemiology and Biostatistics, School of Public Health, Anhui Medical University, Anhui Provincial Laboratory of Inflammatory and Immune Diseases, Hefei, China

**Keywords:** rheumatoid arthritis, autoimmune disease, vitamin D metabolic pathway, methylation, single nucleotide polymorphisms

## Abstract

Abnormal vitamin D metabolism is involved in the pathogenesis of rheumatoid arthritis (RA). In this study, we evaluated the association of single nucleotide polymorphisms (SNPs) and methylation levels in vitamin D metabolic pathway genes with RA susceptibility. Ten SNPs in vitamin D metabolic pathway genes (*CYP2R1*, *CYP24A1*, *VDR*, *CYP27B1*) were genotyped in 477 RA patients and 496 controls by improved multiple ligase detection reaction (iMLDR). The methylation levels of the promoter regions of these genes were detected in 122 RA patients and 123 controls using Illumina Hiseq platform. We found that the *CYP2R1* rs1993116 GA genotype, *CYP27B1* rs4646536 GA genotype, rs4646536 A allele frequencies were significantly increased in RA patients when compared to controls. The decreased risk of rs1993116, rs4646536 was found under the dominant mode in RA patients. However, no significant association was found between *CYP2R1* rs7936142, rs12794714, *CYP24A1* rs2762934, rs6068816, rs2296239, rs2296241, *VDR* rs11574129, rs3847987 polymorphism, and RA susceptibility. The *VDR*, *CYP27B1* methylation levels in RA patients were significantly lower than those in controls, while *CYP2R1*, *CYP24A1* methylation levels were not associated with RA. There were no statistical associations between *CYP2R1*, *CYP24A1*, *VDR*, *CYP27B1* methylation levels and their respective genotype in RA patients. In addition, plasma 25OHD level in RA patients was significantly lower than that in healthy controls. In summary, our results showed that *CYP2R1*, *CYP27B1* genetic variations were associated with the genetic background of RA, while altered *VDR*, *CYP27B1* methylation levels were related to the risk of RA.

## Introduction

Rheumatoid arthritis (RA) is a common autoimmune, chronic inflammatory disease, with a prevalence of 1% around the world (1). The disease is characterized primarily by affecting peripheral joints of the hands, wrists, and feet and can eventually lead to the accumulation of joint damage and irreversible disability in patients (2). At present, the pathogenesis of RA has not been fully elucidated, and individual progress is highly variable during the development of RA; thence, early diagnosis and treatment to maximize the chance of inducing remission is important to prevent permanent disabling joint damage. Many studies have explored the influence of genetic susceptibilities, epigenomic features, abnormal immune response, and environmental factors on the onset, disease activity, and prognosis of RA (3–5). A large number of genes/loci and even some specific variants underlying RA in different races have been identified, with different study protocols like genome-wide association study (GWAS), and candidate gene approaches, while these genes/loci only account for a fraction of phenotypic variance in RA (6, 7). Therefore, it is necessary to study whether immune-modulating gene variation is related to RA susceptibility.

With the development of molecular biology, the regulation of epigenetic changes in gene expression and disease progression has received more and more attention (8). DNA methylation, which is an important key epigenetic trait, involves the addition of a methyl group to the cytosine of CpG dinucleotides and is associated with many biological processes, including X-chromosome inactivation, genomic imprinting, aging, and canceration (9). According to recent studies, epigenetic changes by DNA methylation were dynamic, individual, and highly important in inflammatory processes, and influencing mechanisms of DNA methylation such as DNA methyltransferases activity could directly affect the RA development and might be a very promising therapeutic target for RA (10, 11).

In another context, the association between DNA methylation and inflammation-regulating immune pathways might play a significant role in the pathogenesis of RA (11). Recent studies had suggested that the vitamin D metabolic pathway might be a potential contributor to RA (12). *In vitro*, vitamin D metabolites modulate inflammation by altering the function of T helper and regulatory T cell (12, 13). *In vivo*, vitamin D metabolites including the 25-hydroxyvitamin D (25OHD, the main circulating metabolite) and 1,25-dihydroxyvitamin D (1,25(OH)2D, the active form of vitamin D) had been reported to be associated with RA disease progression, and vitamin D supplementation might have beneficial effects for RA patients (14–16). Moreover, some studies also analyzed the correlation between vitamin D metabolism gene polymorphisms and RA, although the results were inconsistent (17, 18).

The methylation levels of several key genes in the vitamin D metabolic pathway were found to associate with the risk and prognosis of tuberculosis (19). However, the role of vitamin D metabolic pathway gene methylation levels in RA had not been systematically studied. Thus, we performed this molecular epidemiological study to explore the effect of genetic variation, aberrant DNA methylation in four key vitamin D metabolic pathway genes (*CYP2R1*, *CYP24A1*, *VDR*, *CYP27B1*) on the risk of RA in a Chinese population.

## Materials and Methods

### RA Patients and Normal Controls

In this case-control study, a total of 973 subjects including 477 RA patients and 496 normal controls were consecutively included to explore the relationship between *CYP2R1*, *CYP24A1*, *VDR*, *CYP27B1* gene polymorphisms and RA susceptibility. Then, 122 RA patients and 123 normal controls were enrolled to detect their promoter methylation levels. The RA patients’ diagnosis was based on the 1987 American College of Rheumatology revised criteria (20), and peripheral blood samples and clinical information of all subjects were obtained from Anhui Provincial Laboratory of Inflammatory and Immune Diseases. The clinical data [including anticyclic citrullinate peptide (anti-CCP), rheumatoid factor (RF), *etc.*] and drug treatment (including glucocorticoid, methotrexate) of RA patients were collected. The normal controls did not have a history of inflammatory/autoimmune diseases or cancer.

### SNP Selection

We first sought the genotype data of Han Chinese people in Beijing from CHBS_1000g and Ensembl Genome Browser 85. Then, we selected the tag SNPs, capturing all the common SNPs located in the chromosome locus transcribed into vitamin D metabolic pathway genes (*CYP2R1*, *CYP24A1*, *VDR*, *CYP27B1*) and their flanking 2000 bp region. The selection was performed with Haploview 4.0 software (Cambridge, MA, USA). Moreover, the existing studies on the association between *CYP2R1*, *CYP24A1*, *VDR*, *CYP27B1* gene polymorphism and RA susceptibility were also reviewed to search for significant SNP. Finally, we selected one tagSNP (rs4646536) in *CYP27B1*, three tagSNPs (rs12794714, rs7936142, rs1993116) in *CYP2R1*, four tagSNPs (rs2762934, rs6068816, rs2296239, rs2296241) in *CYP24A1*, and two tagSNPs (rs11574129, rs3847987) in *VDR* for genotyping in the present study. Above SNPs accorded with MAF ≥ 0.05 in CHB and *r^2^* threshold > 0.8.

### Genotyping and Methylation Analysis

The genomic DNA was extracted from the peripheral blood leukocytes by the Flexi Gene-DNA Kit (Qiagen, Valencia, CA, USA). Improved multiple ligase detection reaction (iMLDR) genotyping assay was used for genotyping with technical support from the Center for Genetic & Genomic Analysis, Genesky Biotechnologies (Inc., Shanghai). Those individuals with 100% genotyping success rate for the above SNPs were included for final analysis.

The methylation level of vitamin D metabolic pathway genes was detected using MethylTarget^®^ with technical support from the Center for Genetic & Genomic Analysis, Genesky Biotechnologies (Inc., Shanghai). We sequenced the CpG islands in the promoter region of *CYP2R1*, *CYP24A1*, *VDR*, *CYP27B1* by the Illumina Hiseq platform. Primers were designed to amplify the specific sites of interest from the bisulfite-converted DNA (**Table 1**), and the mean methylation level of all CpG sites on the fragment was calculated as the methylation level of the specific sites of each gene.

**Table 1:** The primers of specific sites in each gene.

Gene	Fragment	Forward primer	Reverse primer
CYP24A1	CYP24A1_1	AGGTTGGGGGTATTTGGTTTTT	CCCRAAAATAACCCCCAAAA
	CYP24A1_2	TTTTTGTTGATGGGGGAGTTT	CAACCCCTACRACCAATACAAAA
	CYP24A1_3	GAGGYGGGAGGAGGGAAAG	AAAATCAACAACCCRTAACCTTCTTT
	CYP24A1_4	GGGAGAGGGGTTTTGGTATT	ACACCTAAACTCRCCATACCTACTAAAAAC
CYP27B1	CYP27B1_1	GGGTTTTTGGGGGTAGAGA	ATCCRCTCCCCCAAATACAA
CYP2R1	CYP2R1_1	TTTGTAGGGGGAGTTTYGTTTTT	ACCTACTATTAACCATCTAAAACTCAAAAC
	CYP2R1_2	AAAATAAAATAGGTGAGTTTTGTTTTAGG	AATAACTCATTTAAAACTCATAACCAACC
VDR	VDR_1	GATTAGGGAAGTTGAGATTTAGTTTTT	AAAAACTCAACCTAATCCCACAAA
	VDR_2	AGGTGTTGGGTTGTTTTTGTTTG	ACTTCAACTTTCTCAAACCTCAATACC

### Enzyme-Linked Immunosorbent Assay

In this study, an additional 2 ml peripheral blood was collected from 84 RA patients and 84 normal patients by EDTA anticoagulant tube, and then plasma was obtained by Ficoll-Hypaque density gradient centrifugation. Plasma 25OHD level was determined by ELISA kits (MyBioSource Inc., USA), and the result was expressed as nanograms per milliliter.

### Statistical Analysis

Statistical analysis was conducted with the SPSS 23.0 (Armonk, NY: IBM Corp, USA). Hardy-Weinberg equilibrium test was performed in normal controls with Chi-square (*χ^2^*). Logistic regression analysis was used to calculate the association between genotype, allele distribution frequencies of each SNP and RA risk. Two genetic models (dominant model, recessive model) were also analyzed, and SHEsis software was used to calculate haplotype analysis. The methylation levels of candidate genes were shown as median value and interquartile range, and the differences in methylation levels between two groups and three groups were analyzed by Mann-Whitney *U* test and Kruskal-Wallis *H* test, respectively. The diagnostic value of candidate gene methylation levels in RA patients was calculated by receiver operating characteristic (ROC) analysis. *P* value < 0.05 was considered as statistically significant.

## Results

### Association of Vitamin D Metabolic Pathway Gene Polymorphisms With RA Susceptibility

In this step, we included 477 RA patients and 496 controls. The RA patients consisted of 389 females and 88 males, with an average age of 52.70 ± 12.25 years, and 384 females and 112 males were enrolled in controls with a mean age of 50.61 ± 14.76 years. **Table 2** shows the allele and genotype frequency distributions of all SNPs in RA patients and normal controls, and all SNPs were conformed to Hardy Weinberg equilibrium in controls.

**Table 2:** Genotypes and allele frequencies of vitamin D metabolic pathway genes in RA patients and controls.

SNP	Analyze model		RA (n = 477)	Control (n = 496)	*P* value	*OR* (95% *CI*)
*CYP2R1*						
rs7936142	Genotypes	TT	6 (1.26)	7 (1.41)	0.849	1.112 (0.370,3.341)
		AT	102 (21.38)	102 (20.56)	0.763	0.953 (0.700,1.299)
		AA	369 (77.36)	387 (78.02)	Reference	
	Alleles	T	114 (11.95)	116 (11.69)	0.861	0.976 (0.741,1.285)
		A	840 (88.05)	876 (88.31)	Reference	
	Dominant model	AA	369 (77.36)	387 (78.02)	0.803	1.039 (0.768,1.405)
		AT+TT	108 (22.64)	109 (21.98)	Reference	
	Recessive model	TT	6 (1.26)	7 (1.41)	0.835	1.124 (0.375,3.368)
		AA+AT	471 (98.74)	489 (98.59)	Reference	
rs12794714	Genotypes	AA	54 (11.32)	66 (13.31)	0.280	1.254 (0.832,1.891)
		GA	225 (47.17)	237 (47.78)	0.573	1.081 (0.825,1.415)
		GG	198 (41.51)	193 (38.91)	Reference	
	Alleles	A	333 (34.91)	369 (37.20)	0.293	1.105 (0.918,1.329)
		G	621 (65.09)	623 (62.80)	Reference	
	Dominant model	GG	198 (41.51)	193 (38.91)	0.409	0.898 (0.695,1.160)
		AA+GA	279 (58.49)	303 (61.09)	Reference	
	Recessive model	AA	54 (11.32)	66 (13.31)	0.347	1.202 (0.819,1.765)
		GG+GA	423 (88.68)	430 (86.69)	Reference	
rs1993116	Genotypes	AA	69 (14.47)	68 (13.71)	0.289	0.810 (0.548,1.196)
		GA	233 (48.85)	215 (43.35)	**0.047**	0.758 (0.577,0.996)
		GG	175 (36.69)	213 (42.94)	Reference	
	Alleles	A	371 (38.89)	351 (35.38)	0.110	0.860 (0.716,1.034)
		G	583 (61.11)	641 (64.62)	Reference	
	Dominant model	GG	175 (36.69)	213 (42.94)	**0.047**	1.299 (1.004,1.680)
		AA+GA	302 (63.31)	283 (57.06)	Reference	
	Recessive model	AA	69 (14.47)	68 (13.71)	0.735	0.939 (0.655,1.348)
		GG+GA	408 (85.53)	428 (86.29)	Reference	
*CYP24A1*						
rs2296239	Genotypes	CC	62 (13.00)	63 (12.70)	0.897	0.974 (0.649,1.460)
		CT	232 (48.64)	242 (48.79)	0.997	0.999 (0.762,1.311)
		TT	183 (38.36)	191 (38.51)	Reference	
	Alleles	C	356 (37.32)	368 (37.10)	0.920	0.991 (0.824,1.191)
		T	598 (62.68)	624 (62.90)	Reference	
	Dominant model	TT	183 (38.36)	191 (38.51)	0.963	1.006 (0.777,1.303)
		CC+CT	294 (61.64)	305 (61.49)	Reference	
	Recessive model	CC	62 (13.00)	63 (12.70)	0.890	0.974 (0.669,1.418)
		TT+CT	415 (87.00)	433 (87.30)	Reference	
rs2296241	Genotypes	AA	94 (19.71)	87 (17.54)	0.394	0.853 (0.593,1.229)
		GA	229 (48.01)	242 (48.79)	0.858	0.975 (0.734,1.294)
		GG	154 (32.29)	167 (36.67)	Reference	
	Alleles	A	417 (43.71)	416 (41.94)	0.429	0.930 (0.777,1.113)
		G	537 (56.29)	576 (58.06)	Reference	
	Dominant model	GG	154 (32.29)	167 (33.67)	0.646	1.065 (0.815,1.391)
		AA+GA	323 (67.71)	329 (66.33)	Reference	
	Recessive model	AA	94 (19.71)	87 (17.54)	0.386	0.867 (0.627,1.197)
		GG+GA	383 (80.29)	409 (82.46)	Reference	
rs2762934	Genotypes	AA	5 (1.05)	7 (1.41)	0.644	1.314 (0.413,4.176)
		GA	106 (22.22)	99 (19.96)	0.403	0.876 (0.644,1.194)
		GG	366 (76.73)	390 (78.63)	Reference	
	Alleles	A	116 (12.16)	113 (11.39)	0.599	0.929 (0.705,1.224)
		G	838 (87.84)	879 (88.61)	Reference	
	Dominant model	GG	366 (76.73)	390 (78.63)	0.477	1.116 (0.825,1.509)
		AA+GA	111 (23.27)	106 (21.37)	Reference	
	Recessive model	AA	5 (1.05)	7 (1.41)	0.609	1.351 (0.426,4.287)
		GG+GA	472 (98.05)	489 (98.59)	Reference	
rs6068816	Genotypes	TT	66 (13.84)	73 (14.72)	0.304	1.153 (0.879,1.513)
		CT	204 (42.77)	225 (45.36)	0.460	1.156 (0.786,1.701)
		CC	207 (43.40)	198 (39.92)	Reference	
	Alleles	T	336 (35.22)	371 (37.40)	0.318	1.099 (0.913,1.322)
		C	618 (64.78)	621 (62.60)	Reference	
	Dominant model	CC	207 (43.40)	198 (39.92)	0.271	0.867 (0.672,1.118)
		TT+CT	270 (56.60)	298 (60.08)	Reference	
	Recessive model	TT	66 (13.84)	73 (14.72)	0.695	1.075 (0.750,1.540)
		CC+CT	411 (86.16)	423 (85.28)	Reference	
*VDR*						
rs3847987	Genotypes	AA	26 (4.45)	25 (5.04)	0.818	0.935 (0.527,1.658)
		CA	168 (35.22)	180 (36.29)	0.762	1.042 (0.798,1.360)
		CC	283 (59.33)	291 (58.67)	Reference	
	Alleles	A	220 (23.06)	230 (23.19)	0.948	1.007 (0.816,1.243)
		C	734 (76.94)	762 (76.81)	Reference	
	Dominant model	CC	283 (59.33)	291 (58.67)	0.834	0.973 (0.754,1.256)
		AA+CA	194 (40.67)	205 (41.33)	Reference	
	Recessive model	AA	26 (5.45)	25 (5.04)	0.774	0.921 (0.524,1.618)
		CC+CA	451 (94.55)	471 (94.96)	Reference	
rs11574129	Genotypes	GG	19 (3.98)	18 (3.63)	0.866	0.944 (0.487,1.832)
		GA	132 (27.67)	151 (30.44)	0.357	1.140 (0.862,1.508)
		AA	326 (68.34)	327 (65.93)	Reference	
	Alleles	G	170 (17.82)	187 (18.85)	0.557	1.071 (0.851,1.348)
		A	784 (82.18)	805 (81.15)	Reference	
	Dominant model	AA	326 (68.34)	327 (65.93)	0.423	0.896 (0.686,1.171)
		GG+GA	151 (31.66)	169 (34.07)	Reference	
	Recessive model	GG	19 (3.98)	18 (3.63)	0.773	0.908 (0.470,1.752)
		AA+GA	458 (96.02)	478 (96.37)	Reference	
*CYP27B1*						
rs4646536	Genotypes	AA	72 (15.09)	64 (12.90)	0.085	0.710 (0.480,1.048)
		GA	227 (47.59)	209 (42.14)	**0.027**	0.735 (0.560,0.965)
		GG	178 (37.32)	223 (44.96)	Reference	
	Alleles	A	371 (38.89)	337 (33.97)	**0.024**	0.809 (0.672,0.973)
		G	583 (61.11)	655 (66.03)	Reference	
	Dominant model	GG	178 (37.32)	223 (44.96)	**0.016**	1.372 (1.062,1.773)
		AA+GA	299 (62.68)	273 (55.04)	Reference	
	Recessive model	AA	72 (15.09)	64 (12.90)	0.325	0.833 (0.580.1.198)
		GG+GA	405 (84.91)	432 (87.10)	Reference	

Bold value means P < 0.05.

We found that *CYP2R1* rs1993116 GA genotype frequency was significantly higher in RA patients than that in normal controls (GA *versus* GG: *P* = 0.047), while a decreased risk of rs1993116 was observed under the dominant mode (GG *versus* AA+GA: *P* = 0.047). In the *CYP27B1* gene, the rs4646536 GA genotype and A allele frequencies were significantly increased in RA patients in comparison to normal controls (GA *versus* GG: *P* = 0.027; A *versus* G: *P* = 0.024, respectively). Moreover, a decreased risk of rs4646536 variant was found under dominant mode (GG *versus* AA+GA: *P* = 0.016). However, there was no significant difference in allele and genotype distribution of the *CYP2R1* rs7936142 and rs12794714 between RA patients and normal controls (all *P*>0.05). Similarly, *CYP24A1* gene rs2762934, rs6068816, rs2296239, rs2296241, and *VDR* gene rs11574129, rs3847987 polymorphisms were not significantly associated with RA.

To explore the potential relationship between *CYP2R1*, *CYP24A1*, *VDR*, and *CYP27B1* genetic variation and anti-CCP, RF status in RA patients, we also conducted a case-only analysis (**Table 3**). We found that allele and genotype frequencies of all SNPs did not have statistically significant differences between anti-CCP-positive RA patients and anti-CCP-negative RA patients, as well as RA patients with RF-positive and with RF-negative.

**Table 3:** Association between vitamin D metabolic pathway gene polymorphisms and anti-CCP, RF in RA patients.

SNPs	Allele	Clinical features	Group	Genotypes			*P* value	Alleles		*P* value
	(M/m)			MM	Mm	mm		M	m	
*CYP2R1*										
rs7936142	A/T	anti-CCP	Positive	274	72	5	0.569	620	82	0.613
			Negative	62	16	0		140	16	
		RF	Positive	291	81	6	0.518	663	93	0.473
			Negative	62	16	0		140	16	
rs12794714	G/A	anti-CCP	Positive	143	172	36	0.082	458	244	0.185
			Negative	30	33	15		93	63	
		RF	Positive	156	178	44	0.888	490	266	0.636
			Negative	30	38	10		98	58	
rs1993116	G/A	anti-CCP	Positive	124	173	54	0.762	421	281	0.510
			Negative	31	36	11		98	58	
		RF	Positive	144	177	57	0.139	465	291	0.770
			Negative	24	46	8		94	62	
*CYP24A1*										
rs2296239	T/C	anti-CCP	Positive	133	176	42	0.779	442	260	0.973
			Negative	31	36	11		98	58	
		RF	Positive	146	186	46	0.945	478	278	0.924
			Negative	29	40	9		98	58	
rs2296241	G/A	anti-CCP	Positive	106	175	70	0.157	387	315	0.189
			Negative	32	31	15		95	61	
		RF	Positive	122	179	77	0.599	423	333	0.401
			Negative	27	39	12		93	63	
rs2762934	G/A	anti-CCP	Positive	267	80	4	0.789	614	88	0.572
			Negative	62	15	1		139	17	
		RF	Positive	287	87	4	0.614	661	95	0.422
			Negative	63	14	1		140	16	
rs6068816	C/T	anti-CCP	Positive	151	152	48	0.593	454	248	0.532
			Negative	38	29	11		105	51	
		RF	Positive	167	162	49	0.506	496	260	0.414
			Negative	33	31	14		97	59	
*VDR*										
rs3847987	C/A	anti-CCP	Positive	211	123	17	0.353	545	157	0.165
			Negative	40	33	5		113	43	
		RF	Positive	228	131	19	0.216	587	169	0.082
			Negative	39	33	6		111	45	
rs11574129	A/G	anti-CCP	Positive	239	100	12	0.897	578	124	0.645
			Negative	51	24	3		126	30	
		RF	Positive	260	104	14	0.675	624	132	0.366
			Negative	50	24	4		124	32	
*CYP27B1*										
		
rs4646536	G/A	anti-CCP	Positive	132	162	57	0.528	426	276	0.621
			Negative	29	40	9		98	58	
		RF	Positive	143	178	57	0.949	464	292	0.794
			Negative	28	38	12		94	62	

### Haplotype Analysis

We constructed the haplotypes of *CYP2R1*, *CYP24A1*, *VDR* through SHEsis software and analyzed the relationship between these haplotypes and RA susceptibility. Seven main haplotypes (CAAC, CAGC, CGGC, CGGT, TAGC, TGGC, TGGT) for *CYP24A1*, three main haplotypes (AAG, AGA, AGG) for *CYP2R1*, and three main haplotypes (AA, AG, CA) for *VDR* were detected by SHEsis in this study (**Table 4**). The results demonstrated that there was no statistically significant difference in the frequency distribution of the above haplotypes between RA patients and normal controls (all *P*>0.05).

**Table 4:** Haplotype analysis of *CYP2R1, CYP24A1, VDR* in RA patients and controls.

Haplotype	RA [n(%)]	Controls [n(%)]	*P* value	*OR* (95% CI)
*CYP2R1* rs7936142-rs12794714-rs1993116				
AAG	332.99 (34.9)	368.99 (37.2)	0.293	0.905 (0.752,1.090)
AGA	370.99 (38.9)	350.99 (35.4)	0.109	1.162 (0.967,1.397)
AGG	250.01 (26.2)	272.01 (27.4)	0.545	0.940 (0.769,1.149)
*CYP24A1* rs2296239-rs2296241-rs2762934-rs6068816				
CAAC	85.12 (8.9)	76.67 (7.7)	0.387	1.153 (0.835,1.593)
CAGC	74.28 (7.8)	85.27 (8.6)	0.457	0.884 (0.638,1.224)
CGGC	95.08 (10.0)	102.59 (10.3)	0.706	0.945 (0.703,1.270)
CGGT	60.57 (6.3)	60.03 (6.1)	0.846	1.037 (0.717,1.501)
TAGC	224.08 (23.5)	218.03 (2.5)	0.525	1.072 (0.865,1.328)
TGGC	128.85 (13.5)	124.54 (12.6)	0.610	1.071 (0.822,1.397)
TGGT	232.19 (24.3)	256.14 (25.8)	0.348	0.905 (0.736,1.114)
*VDR* rs3847987- rs11574129				
AA	50.00 (5.2)	40.00 (4.3)	0.348	1.221 (0.804,1.854)
AG	170.00 (17.8)	187.00 (18.9)	0.557	0.933 (0.742,1.175)
CA	734.00 (76.9)	762.00 (76.8)	0.948	1.007 (0.816,1.243)

Frequency < 0.03 in both controls and RA patients has been dropped.

### The Methylation Levels of Vitamin D Metabolic Pathway Genes in RA Patients and Normal Controls

In this step, the RA group included 100 females and 22 males, with a mean age of 52.61±13.05 years, and the control group included 82 females and 41 males, with an average age of 46.93±14.29 years. The methylation levels of specific sites between RA patients and controls are shown in **Table 5**. The results demonstrated that CYP24A1_1, CYP27B1_1, and VDR_1 methylation levels were significantly lower in RA patients than that in normal controls (*P*=0.032, *P*<0.001, *P*<0.001, respectively).

**Table 5:** Methylation levels of specific sites between RA patients and controls.

Group	RA patients (n = 122)	Controls (n = 123)	*P* value
CYP2R1_1	0.0072 (0.0068,0.0076)	0.0072 (0.0065,0.0077)	0.708
CYP2R1_2	0.0071 (0.0066,0.0079)	0.0072 (0.0065,0.0079)	0.960
CYP24A1_1	0.0136 (0.0120,0.0155)	0.0143 (0.0127,0.0164)	**0.032**
CYP24A1_2	0.0615 (0.0560,0.0700)	0.0640 (0.0554,0.0737)	0.228
CYP24A1_3	0.0314 (0.0267,0.0376)	0.0318 (0.0271,0.0384)	0.607
CYP24A1_4	0.0662 (0.0577,0.0774)	0.0685 (0.0613,0.0791)	0.206
VDR_1	0.0316 (0.0260,0.0378)	0.0357 (0.0311,0.0421)	**<0.001**
VDR_2	0.0121 (0.0114.0.0129)	0.0121 (0.0108,0.0130)	0.516
CYP27B1_1	0.0307 (0.0263,0.0373)	0.0345 (0.0308,0.0412)	<0.001

Bold value means P < 0.05.

**Figure 1:**
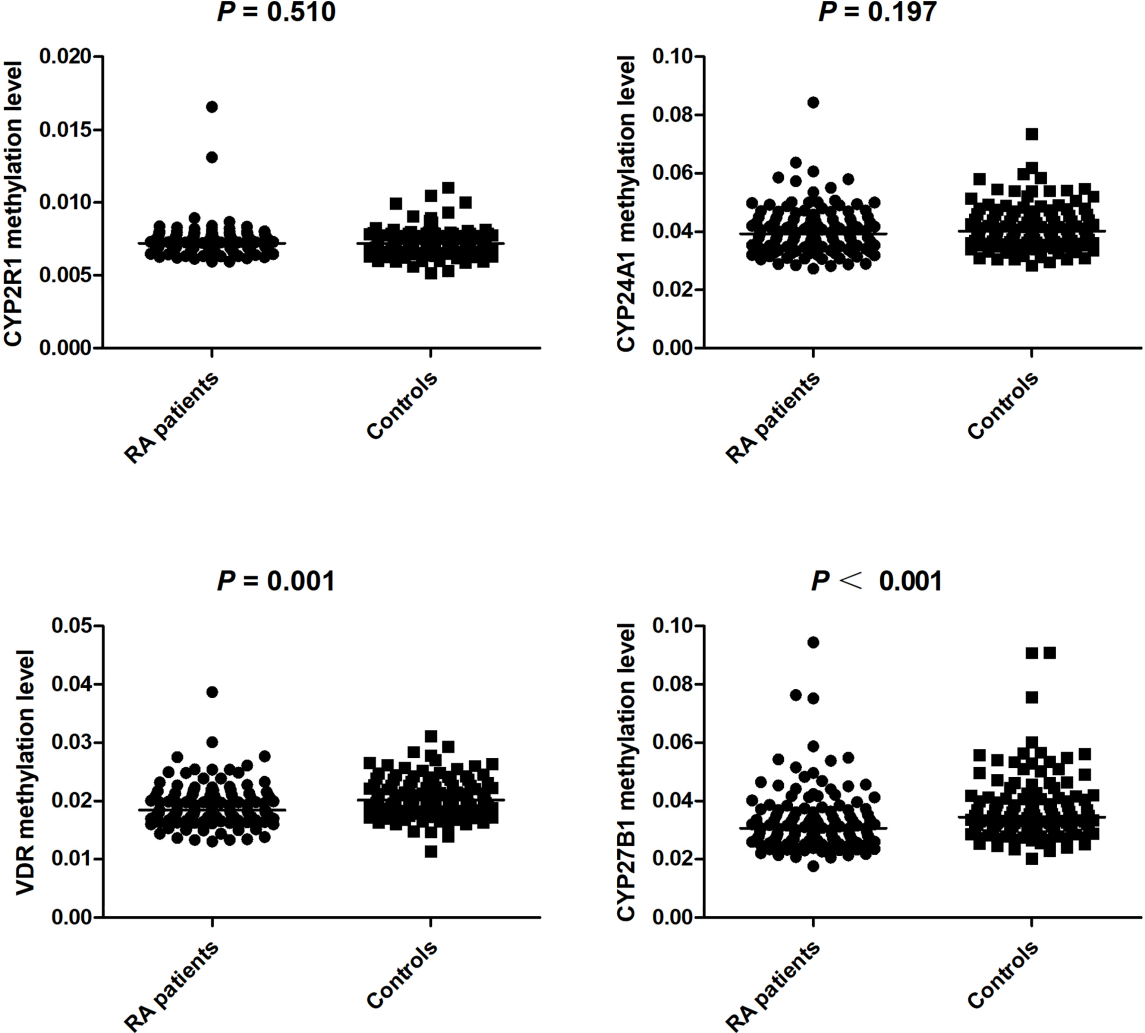
The methylation levels of CYP2R1, CYP24A1, VDR, CYP27B1 between RA patients and controls.

**Figure 2:**
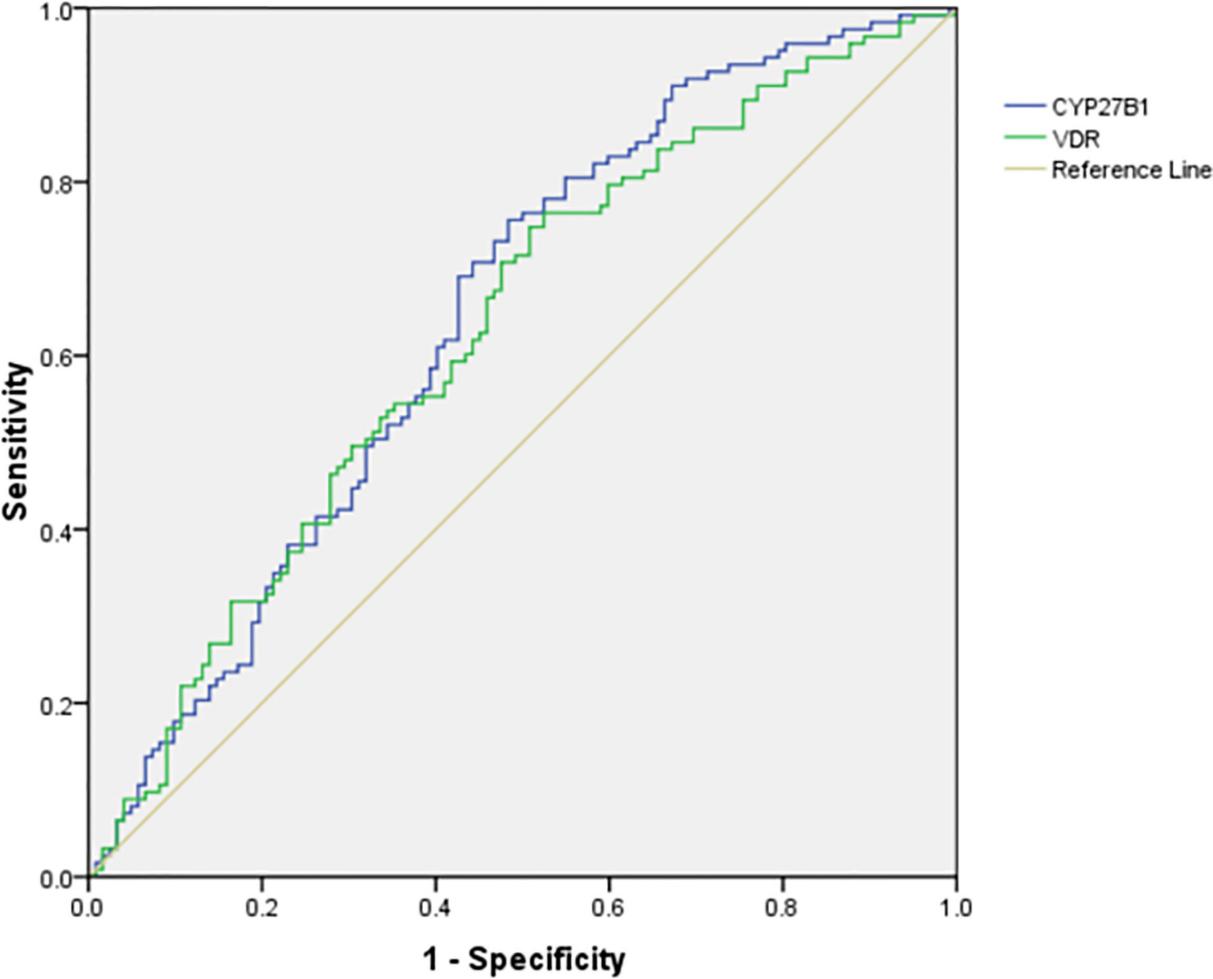
The diagnostic accuracy of VDR and CYP27B1 methylation level in RA.

We further calculated the cumulative methylation levels of each gene by calculating the mean methylation levels of all CpG sites on the included fragments. The methylation levels of VDR and CYP27B1 were significantly reduced when compared to normal controls, while the differences in CYP2R1 and CYP24A1 methylation levels between RA patients and controls were not statistically significant (**Figure 1**). The diagnostic value of VDR and CYP27B1 methylation levels for RA diagnosis was also assessed, and the AUCs of VDR and CYP27B1 were 0.628 (0.559–0.698) and 0.645 (0.575–0.714), respectively (**Figure 2**). Moreover, the optimal cutoff value of VDR for RA diagnosis was 0.018, and the corresponding sensitivity and specificity were 74.8 and 49.2%, respectively. The optimal cutoff value of CYP27B1 for RA diagnosis was 0.031, and the corresponding sensitivity and specificity were 75.6 and 51.2%, respectively.

We also analyzed the influences of the main drug treatment on the methylation levels of these genes, and there were no significant associations regarding CYP24A1, CYP27B1, CYP2R1, and VDR methylation levels between RA patients being treated with glucocorticoid and without, as well as the patients being treated with methotrexate and without (**Table 6**). In addition, the results revealed that CYP24A1, CYP27B1, CYP2R1, and VDR methylation levels were not associated with anti-CCP, RF in RA patients (**Table 6**). Nevertheless, the CYP2R1 methylation level was positively associated with erythrocyte sedimentation rate (ESR) and C-reactive protein (CRP) in RA patients (*P* = 0.003, *P* = 0.018, respectively) (**Table 7**). No significant correlations were observed about CYP27B1, CYP2R1, and VDR methylation levels and ESR and CRP in RA patients.

**Table 6:** The association between CYP24A1, CYP27B1, CYP2R1, VDR methylation levels and antibody, drug treatment in RA patients.

Group	N	CYP2R1 methylation level	*P* value	CYP24A1 methylation level	*P* value	VDR methylation level	*P* value	CYP27B1 methylation level	*P* value
Antibody									
Anti-CCP			0.460		0.930		0.815		0.414
+	88	0.0073 (0.0069,0.0078)		0.0399 (0.0351,0.0451)		0.0189 (0.0171,0.0213)		0.0310 (0.0264,0.0387)	
−	14	0.0072 (0.0065,0.0076)		0.0393 (0.0350,0.0471)		0.0193 (0.0168,0.0250)		0.0345 (0.0271,0.0402)	
RF			0.266		0.556		0.504		0.770
+	99	0.0072 (0.0068,0.0076)		0.0396 (0.0348,0.0451)		0.0186 (0.0170,0.0212)		0.0312 (0.0263,0.0385)	
−	17	0.0075 (0.0070,0.0078)		0.0386 (0.0340,0.0454)		0.0174 (0.0167,0.0209)		0.0305 (0.0268,0.0354)	
Drug treatment									
glucocorticoid			0.794		0.859		0.067		0.924
+	68	0.0072 (0.0067,0.0077)		0.0305 (0.0261,0.0382)		0.0177 (0.0164,0.0207)		0.0305 (0.0261,0.0382)	
−	40	0.0072 (0.0068,0.0076)		0.0391 (0.0348,0.0450)		0.0192 (0.0177,0.0217)		0.0311 (0.0267,0.0362)	
Methotrexate			0.300		0.877		0.690		0.877
+	38	0.0072 (0.0065,0.0075)		0.0390 (0.0347,0.0444)		0.0187 (0.0167,0.0203)		0.0300 (0.0262,0.0370)	
−	70	0.0072 (0.0068,0.0078)		0.0394 (0.0345,0.0428)		0.0184 (0.0172,0.0212)		0.0309 (0.0266,0.0374)	

**Table 7:** The relationship between CYP24A1, CYP27B1, CYP2R1, VDR methylation levels and ESR and CRP in RA patients.

Parameters	*CYP2R1*		*CYP24A1*		*VDR*		*CYP27B1*	
	*r_s_*	*P* value	*r_s_*	*P* value	*r_s_*	*P* value	*r_s_*	*P* value
ESR	0.338	**0.003**	0.027	0.822	0.110	0.353	0.002	0.985
CRP	0.274	**0.018**	0.091	0.442	0.053	0.657	0.169	0.151

Bold value means P < 0.05.

### Associations Between Vitamin D Metabolic Pathway Gene Polymorphisms With Their Methylation Levels in RA Patients

To explore the associations between the genotype frequencies of *CYP2R1*, *CYP24A1*, *VDR*, and *CYP27B1* genes and their methylation levels among RA patients, we included 122 RA patients for analysis. The results showed no statistical associations between *CYP2R1*, *CYP24A1*, *VDR*, *CYP27B1* methylation levels and their respective genotype in RA patients (**Table 8**).

**Table 8:** Association between vitamin D metabolic pathway gene polymorphisms with their methylation levels in RA patients.

CYP2R1 SNP	Genotype	Number	CYP2R1 methylation level	P value
rs7936142	TT	1	0.0069	0.528
	AT	27	0.0072 (0.0069,0.0079)	
	AA	94	0.0072 (0.0068,0.0076)	
rs12794714	AA	12	0.0074 (0.0068,0.0079)	0.772
	GA	53	0.0072 (0.0068,0.0078)	
	GG	57	0.0072 (0.0069,0.0076)	
rs1993116	AA	19	0.0071 (0.0068,0.0075)	0.602
	GA	67	0.0072 (0.0068,0.0077)	
	GG	36	0.0073 (0.0069,0.0078)	
*CYP24A1* SNP	Genotype	Number	CYP24A1 methylation level	*P* value
rs2296239	CC	18	0.0363 (0.0318,0.0475)	0.619
	CT	55	0.0382 (0.0347,0.0447)	
	TT	49	0.0396 (0.0355,0.0451)	
rs2296241	AA	20	0.0414 (0.0354,0.0457)	0.393
	GA	62	0.0381 (0.0334,0.0433)	
	GG	40	0.0412 (0.0350,0.0464)	
rs2762934	AA	2	0.0504 (0.0429,0.0579)	0.290
	GA	25	0.0377 (0.0325,0.0479)	
	GG	95	0.0392 (0.0348,0.0442)	
rs6068816	TT	18	0.0405 (0.0360,0.0474)	0.409
	CT	52	0.0387 (0.0337,0.0438)	
	CC	52	0.0396 (0.0355,0.0462)	
*VDR* SNP	Genotype	Number	*VDR* methylation level	*P* value
rs3847987	AA	6	0.0173 (0.0146,0.0197)	0.132
	CA	41	0.0198 (0.0173,0.0221)	
	CC	75	0.0181 (0.0172,0.0202)	
rs11574129	GG	6	0.0173 (0.0146,0.0197)	0.060
	GA	34	0.0198 (0.0175,0.0225)	
	AA	82	0.0181 (0.0170,0.0203)	
*CYP27B1* SNP	Genotype	Number	CYP27B1 methylation level	*P* value
rs4646536	AA	14	0.0314 (0.0267,0.0384)	0.647
	GA	57	0.0311 (0.0268,0.0372)	
	GG	51	0.0294 (0.0256,0.0372)	

Median (interquartile range).

### Plasma Level of 25OHD From RA Patients and Normal Controls

Finally, plasma 25OHD level was measured in 84 RA patients and 84 normal controls in this study. There were 76 females and 8 males in the RA group, with an average age of 53.15 ± 11.98 years. The control group included 77 females and 7 males, with a mean age of 52.57 ± 9.56 years. We found that the plasma level of 25OHD in RA patients (34.20 ± 5.15 ng/ml) was significantly lower than that in healthy controls (41.09 ± 7.52 ng/ml) (*P*<0.001).

## Discussion

Epidemiological studies had shown a high prevalence of vitamin D deficiency in autoimmune diseases, which could lead to worse disease clinical activity and progression of RA, systemic lupus erythematosus (SLE), and multiple sclerosis (MS) (21). Vitamin D deficiency in patients with autoimmune diseases and general population might be caused by several factors, including skin pigmentation, lack of exposure to sunlight, glucocorticoids use, genetic background, and age (22). Previous studies had identified the potential role of multiple genes, which could regulate vitamin D levels, and suggested that SNPs in these genes (CYP27B1, CYP2R1, VDR, etc.) were related to decreased vitamin D level (17, 23). Therefore, in-depth exploration of the association between vitamin D metabolic pathway gene SNPs and genetic risk of autoimmune diseases was helpful to further reveal the pathogenesis of these diseases. In this study, we analyzed the relationship between 10 SNPs in vitamin D metabolic pathway genes (*CYP2R1*, *CYP24A1*, *CYP27B1*, *VDR*) and RA susceptibility in a Chinese population, and detected the methylation levels of these genes in RA patients.

Among the vitamin D metabolic pathway genes, the presence of SNPs might influence autoimmune disease genetic susceptibility through causing vitamin D deficiency, and modulate the disease activity in type 1 diabetes (TID), SLE, MS. Chen et al. investigated the association of *VDR* polymorphism and genetic risk of SLE in a Chinese population and found that *VDR* rs2228570, rs1544410 polymorphism, and their interaction were all associated with increased SLE risk (24). The role of vitamin D metabolic pathway genes in RA had also been reported in previous studies, such as *CYP2R1* rs10741657 played an effect on vitamin D levels in RA patients (25). In the present study, we found that *CYP2R1* rs1993116, *CYP27B1* rs4646536 polymorphisms were significantly associated with RA susceptibility. In addition to this result, the decreased risk of rs1993116, rs4646536 variant was found to be related to RA risk in dominant mode. Previous studies suggested that rs1993116, rs4646536 variants were closely associated with vitamin D deficiency in human diseases (26, 27). Therefore, we assumed that rs1993116, rs4646536 might be involved in the development of RA by affecting vitamin D status, and the mechanism needed to be validated by more rigorous studies with a larger sample size and different ethnic population. *CYP2R1* rs12794714, *CYP24A1* rs2762934, rs6068816, and *VDR* rs11574129 had been reported to be involved in the genetic background of multiple diseases including diabetic ischemic stroke, gastric cancer, and other diseases (28–31). Unfortunately, this study did not demonstrate a significant association between these SNPs and RA risk. The inconsistencies might be explained by the genetic background of different diseases, sample size, different races, and experimental methods. RA patients could be divided into different genetic subsets according to the antibody status, including RF and anti-CCP (4), while we did not find any significant association with RF and anti-CCP status among RA patients.

In addition to the DNA sequence, genetic information also existed in epigenetic variation, and the role of epigenetic variation in the pathogenesis of diseases should not be overlooked (8, 32). For example, promoter methylation was involved in tumor development by silencing tumor suppressor genes (33). DNA hypomethylation was related to differentiation and proliferation of inflammatory processes and might lead to increased transcription and secretion of inflammatory proteins (34). A previous study was performed to detect the methylation status of lymphatic cells in SLE, RA patients, and found a significant hypomethylation in T cells (11, 35). The role of vitamin D metabolic pathway gene methylation in the development of human disease had been studied, and Wang et al. suggested that the methylation levels of the *CYP24A1*, *CYP27A1*, *CYP27B1*, *CYP2R1*, and *VDR* genes were associated with the risk and prognosis of tuberculosis (19). Another study also showed that *cytochrome P450* gene silencing caused by hypermethylation in the promoter region might affect vitamin D activity (36). In the present study, we found that *VDR* and *CYP27B1* methylation levels of RA patients were significantly decreased in comparison to normal controls, and ROC curves showed that these genes could be potential biomarkers for the diagnosis of RA. Moreover, it was necessary to further explore the combined diagnostic effect of VDR, CYP27B1, and other indicators, to improve the sensitivity and specificity of these indicators in RA diagnosis. We also found that CYP24A1_1 (one fragment) level was decreased in RA patients, and CYP2R1 methylation level was significantly associated with ESR and CRP. These suggested that CYP24A1, CYP2R1 gene might be involved in the disease process of RA, but further verification was needed. The relationship between methylation and genetic variation among individuals had also been reported in previous studies (37). We further explored the associations between the genotype frequencies of *CYP2R1*, *CYP24A1*, *VDR*, *CYP27B1* genes and their methylation levels among RA patients; however, no statistical significance was found.

Methotrexate was the first-line therapy in early RA and was often prescribed in combination with glucocorticoid, hydroxychloroquine, *etc.* Previous studies had suggested that the anti-inflammatory mechanism of low-dose methotrexate treatment used in RA might relate to the inhibition of key enzymes in the purine *de novo* synthesis pathway and release of anti-inflammatory adenosine (38). In addition, methotrexate could inhibit methionine S-adenosyltransferase (MAT), followed by the inhibition of S-adenosyl methionine (SAM) *in vivo* and *in vitro*; moreover, SAM was responsible for the donation of methyl groups required for global DNA methylation (39, 40). Therefore, methotrexate was hypothesized to affect global DNA methylation, and one study found that higher baseline global DNA methylation was associated with methotrexate non-response (40). However, we observed that the use of methotrexate, as well as glucocorticoids did not have any significant influence on the methylation levels of these genes.

In conclusion, our results demonstrated that *CYP2R1* rs1993116, *CYP27B1* rs4646536 polymorphisms might contribute to the genetic predisposition to RA, while *CYP24A1* and *VDR* gene polymorphisms were not associated with RA susceptibility in a Chinese population. The methylation levels of *VDR* and *CYP27B1* genes were significantly related to the risk of RA and might be regarded as auxiliary biomarkers for RA diagnosis. Furthermore, we also found that the plasma vitamin D level in RA patients was significantly reduced, which was consistent with previous studies. The above results implied that it was of great significance to explore the role of vitamin D metabolism abnormality in the pathogenesis of RA. However, there were some limitations in this that study should be acknowledged. First, this was a case-control study, and we were unable to evaluate the relationship between the methylation levels of these genes and disease activity, medications, clinical efficacy of RA patients over a long period. Second, this study did not analyze the potential influence of ethnic background and environmental factors, as well as the interaction between environmental factors and genetic variation, in RA patients. Finally, the genotyping and ELISA tests were not performed in the same samples, and we were unable to further analyze the relationship between gene polymorphism, methylation, and vitamin D level. The precise role of vitamin D metabolic pathway genes in RA development needed to be further explored in repetitive, functional studies in the future.

## Data Availability Statement

The data presented in the study are deposited in the dbSNP repository, accession number 1063300. Further inquiries can be directed to the corresponding authors.

## Ethics Statement

The studies involving human participants were reviewed and approved by the Ethical Committee of the First Affiliated Hospital of USTC. The patients/participants provided their written informed consent to participate in this study.

## Author Contributions

X-ML and T-PZ designed the study. T-PZ and H-ML conducted the experiment. H-ML performed the statistical analyses. QH and LW participated in the collection of samples. T-PZ drafted the manuscript. X-ML and LW contributed to manuscript revision. All authors contributed to the article and approved the submitted version.

## Funding

This work was supported by the Fundamental Research Funds for the Central Universities (WK9110000180, WK9110000148), National Natural Science Foundation of China (81871271), and Anhui Provincial Natural Science Foundation (2108085QH362).

## Conflict of Interest

The authors declare that the research was conducted in the absence of any commercial or financial relationships that could be construed as a potential conflict of interest.

## Publisher’s Note

All claims expressed in this article are solely those of the authors and do not necessarily represent those of their affiliated organizations, or those of the publisher, the editors and the reviewers. Any product that may be evaluated in this article, or claim that may be made by its manufacturer, is not guaranteed or endorsed by the publisher.
